# Dual‐Atom Catalyst Au@S‐rGO for Rapid and Highly Sensitive Electrochemical Detection of Fentanyl in Serum

**DOI:** 10.1002/advs.202500430

**Published:** 2025-03-16

**Authors:** Meng Li, ZhiJin Fan, Qiuxia Gao, Ying He, Anyun Xu, Zhaofeng Gu, Shixiong Wang, Huiping Bai, Yuhui Liao, Ruilin Zhang

**Affiliations:** ^1^ School of Forensic Medicine NHC Key Laboratory of Drug Addiction Medicine Institute for Engineering Medicine Kunming Medical University, Kunming Kunming Yunnan 650500 China; ^2^ School of Material and Energy Institute of International Rivers and Eco‐Security School of Chemical Science and Technology Yunnan University Kunming Yunnan 650091 China

**Keywords:** animal experiments on rats, Au@S‐rGO, electrochemical, fentanyl

## Abstract

Fentanyl is a highly lethal emerging drug that requires urgent rapid trace detection. In this work, high‐sensitivity detection of fentanyl is achieved by Au‐ and S‐doped reduced graphene oxide (Au@S‐rGO). Spherical electron microscopy revealed that Au and S existed in an atomically dispersed state. The possible configurations of S in Au@S‐rGO, as well as the effects of different doping positions on the electron density of Au, are analyzed by Density functional theory (DFT) simulations. The co‐modification of metal and nonmetal atoms significantly improves the catalytic activity of the electrode. The optimal electrode delivers a good linear relationship for fentanyl concentrations from 0.0291 to 38.2 µmol L‐1, with a detection limit of 9.7 nmol L‐1. The recovery rate of fentanyl in human serum ranged from 98.0% to 104%, demonstrating the precision of the sensor in real biological matrices. Furthermore, by employing rats in place of drug addicts, the coincidence rate between the electrochemical test results and the mass spectrometry results was 85.7% ~ 93.6%. Compared with mass spectrometry, the sensor offers faster, simpler, and more cost‐effective onsite detection. In summary, the novel diatomic catalyst design looks excellent for fabricating electrochemical sensors for the rapid detection of fentanyl in real samples.

## Graphical abstract

This study develops an Au/S‐doped reduced graphene oxide (Au@S‐rGO) sensor for rapid fentanyl detection. DFT simulations and structural analysis confirm atomically dispersed Au/S configurations, enhancing catalytic activity. The sensor achieves a 9.7 nmol L^−1^ detection limit, 98–104% recovery in human serum, and 85.7–93.6% consistency with mass spectrometry in rat tests. It offers cost‐effective, on‐site analysis, demonstrating the practical potential for real‐sample applications.

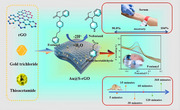

## 1 Introduction

Fentanyl, which has powerful analgesic effects, is widely used as an intravenous anesthetic in clinical procedures.^[^ ^1^ ^]^ However, serious abuse of fentanyl results in high numbers of overdose deaths, with more than 100 000 deaths in 2021 alone.^[^ ^2^, ^3^ ^]^ Fentanyl can also be mixed with other drugs, such as heroin, cocaine, and methamphetamine, resulting in much stronger drug formulations but also increasing the difficulty of trace analysis of fentanyl in complex drug mixtures. Several studies have shown blood concentrations ranging from 3 to 80 nmol L^−1^ in fentanyl abusers and 4 to 50 nmol L^−1^ in people who die from fentanyl‐like drugs.^[^ ^4^, ^5^ ^]^ Therefore, developing portable rapid detection technologies for fentanyl with high sensitivity and accuracy is highly valuable for combating fentanyl drug crime and abuse.

Current detection methods for fentanyl rely primarily on large and expensive instruments, including spectrophotometry, gas chromatography, gas chromatography‐mass spectrometry, liquid chromatography, and liquid chromatography‐mass spectrometry,^[^ ^6^, ^7^, ^8^, ^9^, ^10^, ^11^ ^]^ with mass spectrometry being the standard laboratory method. These techniques necessitate specialized facilities, trained personnel, and considerable time investments, which impede law enforcement's ability to respond swiftly to drug seizures and enforce penalties for abuse.

Electrochemical techniques have been widely used in the onsite screening of various analytes because of their fast response time, high sensitivity, portability, and low cost.^[^ ^12^, ^13^, ^14^, ^15^, ^16^, ^17^ ^]^ Therefore, electrochemical technology has become a crucial tool for in situ analysis and rapid access to chemical information.^[^ ^18^, ^19^, ^20^, ^21^ ^]^ The core component of electrochemical detection relies on the sensing component, the performance of which depends on the composition, structure, and morphology of the electrode material. The ability to construct sensors for quick and accurate identification and capture of target analytes by promoting the redox reaction on the electrode can be achieved by selecting suitable materials.^[^ ^22^, ^23^, ^24^ ^]^

Nanomaterials have greatly advanced the research progress of electrochemical sensors since changes in the structure of nanomaterials may effectively adjust their electrocatalytic performance.^[^ ^25^, ^26^, ^27^ ^]^ Understanding the structure‐performance relationship has allowed the design and synthesis of nanocatalysts with controlled sizes, shapes, compositions, and structures for better catalytic effects. For example, the miniaturization of nanoparticles can effectively improve their catalytic activity, in which an extreme reduction in the size of nanomaterials results in single‐atom nanomaterials.^[^ ^28^, ^29^ ^]^

Single‐atom catalysts exhibit superior activity and selectivity owing to their well‐defined activity checkpoints and strong metal‐support interactions.^[^ ^30^ ^]^ Compared with nanocatalysts, single‐atom catalysts exhibit significantly improved catalytic performance, sparking extensive research interest in energy storage and conversion, environmental management, biosensing, and biomedicine.^[^ ^31^, ^32^, ^33^, ^34^ ^]^ The high utilization rate of single atoms results in unique electrochemical properties with broad application prospects in the field of electrochemical sensing. The limitation of nanomaterial synthesis in terms of size and composition promoted the rapid development of single‐atom catalysts in multidisciplinary research, providing new ideas and methods for constructing electrochemical sensing platforms.

Simultaneously, the structure‐activity relationships between the structure and properties of single atoms have been extensively studied. To further enhance the performance of single atoms, various strategies have been developed, including adjusting the coordination number of single atoms, modifying the interaction between single atoms and carriers, and altering the spacing effect. Among these, the design of diatomic checkpoints and the use of their synergistic effects can effectively improve the performance of single atoms. Some studies have suggested that homonuclear and heteronuclear diatomic checkpoints, such as Fe–Fe, Co–Co, Ni–Ni, Cu–Cu, Fe–Pt, Ru–Ni, Zn–Co, and Ni–Cu, exhibit good performance in catalysis.^[^ ^35^ ^]^ Additionally, the introduction of nonmetallic atoms, such as B, O, P, S, and Se, to modify the coordination environment of the central metal atom can effectively trigger local charge redistribution and regulate the *d*‐band energy level of the metal center due to their unique atomic electronegativity and orbital interaction.^[^ ^36^, ^37^, ^38^, ^39^ ^]^ Qi et al. achieved the simultaneous anchoring of the Fe–Se single atomic site on carbon materials, with charge redistribution regulated through the synergistic effect of the Fe–Se atomic pairs to significantly promote the redox reaction and enable the sensitive detection of hydrogen peroxide.^[^ ^40^ ^]^ The strong electronegativity of the S atom attracts electrons from the Au atom, leading to a shift in the *d*‐band center of the Au atom. This alteration in the electronic structure optimizes the adsorption energy of the Au atoms for the fentanyl reaction intermediates. Moreover, the chemical bonding of the S atom with the carrier material and its interaction with the Au atom can effectively stabilize the single Au atom and prevent aggregation into larger particles during the reaction. Since aggregated Au particles generally have much lower catalytic activity than single‐atom active sites do, maintaining the dispersion of single atoms is crucial for sustaining high catalyst activity.

**Scheme 1:**
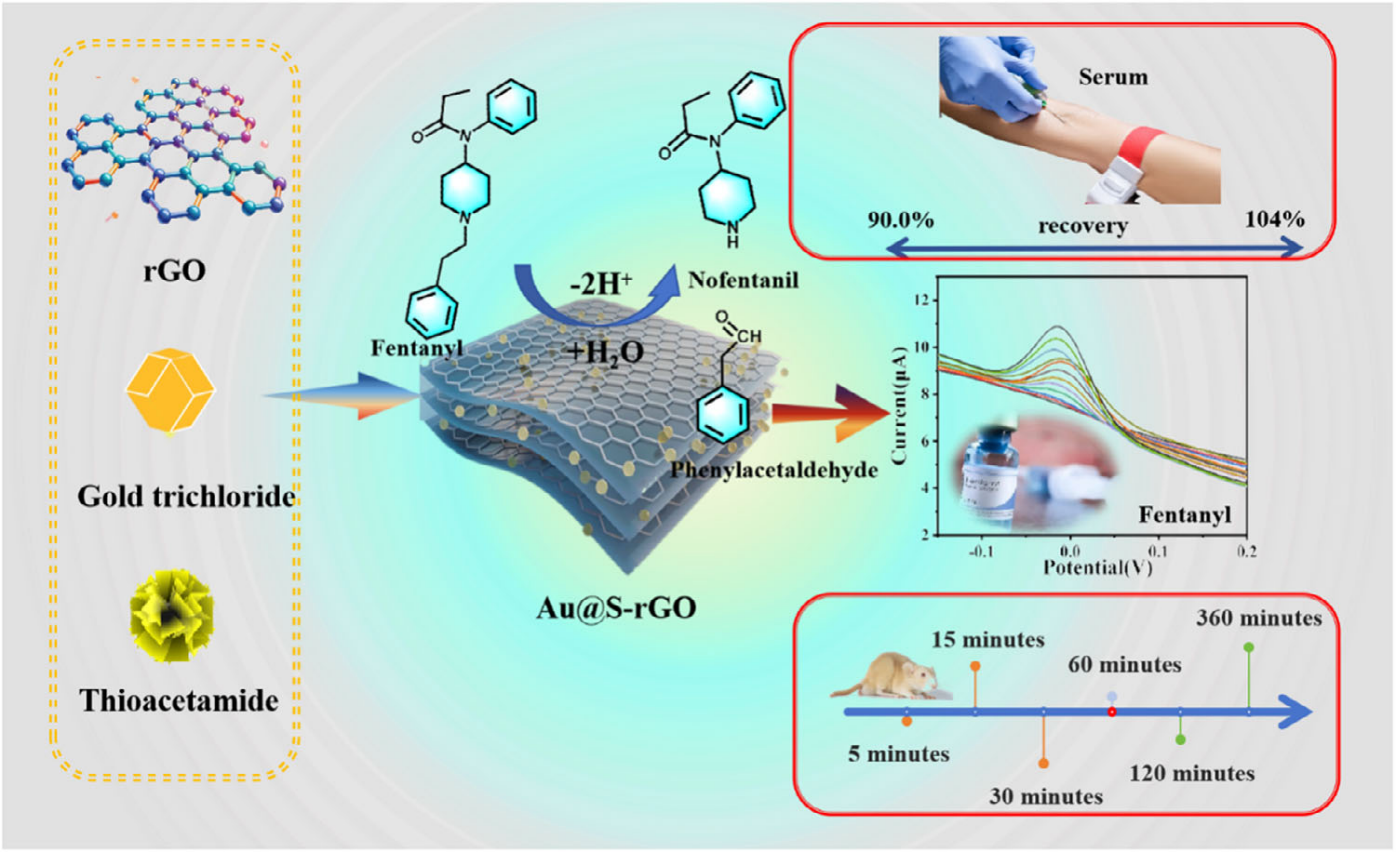
A novel diatomic catalyst facilitates sensitive electrochemical detection of fentanyl in serum samples.

However, research on single atoms in the field of electrochemical sensing is still in its infancy, and research based on diatomic checkpoints is relatively rare. In this study, a new method is developed for the electrochemical detection of fentanyl‐based on diatomic checkpoints constructed by anchoring Au and S atoms into reduced graphene oxide through the solvothermal method (**Scheme** **1**). Compared with the Au single atom, the introduction of the S atom alters the electron density of the Au atom. The simulations reveal changes in the *d*‐band center of the Au element due to the introduced S, enhancing its adsorption capacity and significantly improving the detection effect of the sensor. At the same concentration, the response current is ≈76‐fold greater than that of the glass carbon electrode. The feasibility of the sensor in actual sample detection was tested via rat animal experiments, and the detection results were highly consistent with those of mass spectrometry, indicating the broad prospects of the proposed method in actual field detection.

## 2 Results and Discussion

### 2.1 Morphology Characterization of the Au@S‐rGO/GCE

**Figure 1:**
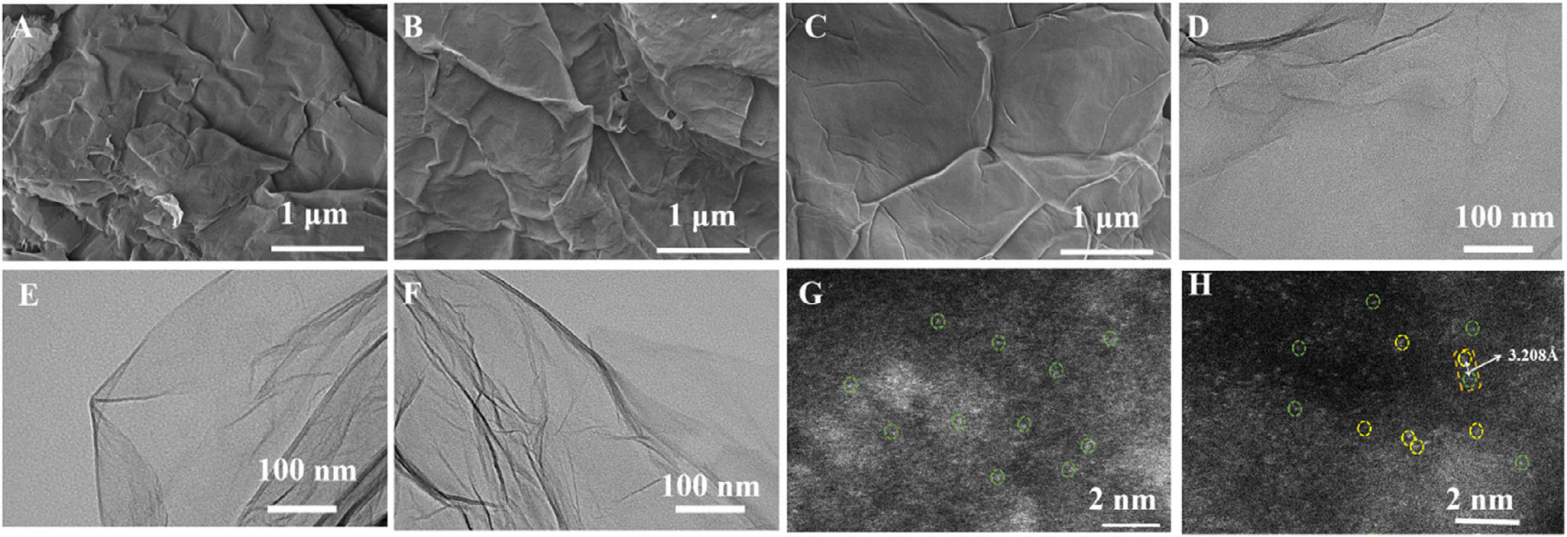
A,D): SEM and TEM images of rGO; B,E,G): SEM, TEM, and STEM images of S‐rGO; C,F,H): SEM, TEM, and STEM images of Au@S‐rGO.

The morphologies and structures of reduced graphene oxide (rGO), single‐atom sulfur‐modified reduced graphene oxide (S‐rGO), and Au‐ and S‐doped reduced graphene oxide (Au@S‐rGO) were characterized by scanning electron microscopy (SEM), transmission electron microscopy (TEM), and high‐angle annular dark field aberration electron microscopy (STEM). **Figure** **1**A–C show SEM images of rGO, S‐rGO, and Au@S‐rGO, respectively. There is no great difference in the morphology of the three materials. rGO has a 2D stacked structure of nanosheets with a relatively large surface area, which is beneficial for providing single‐atom anchoring sites and can promote contact between target molecules and active checkpoints.

Further in‐depth study of the material morphology was carried out via TEM imaging. As depicted in Figure 1D–F, all three materials displayed similar morphologies. No nanoparticles were observed, as shown in Figure 1E,F, indicating that no aggregation of S atoms occurred after the reduction of thioacetamide. S‐rGO provides a strong checkpoint for Au loading, limiting the migration and aggregation of Au. Since no nanoparticles are observed on the rGO surface, S and Au may be distributed as single atoms on the rGO surface.

This suggestion is verified by characterizing S‐rGO and Au@S‐rGO by STEM as effective tools for studying single atoms. Figure 1G,H show STEM images of S‐rGO and Au@S‐rGO, respectively. Both S and Au exist as single atoms on the rGO surface (bright spots). The difference in the atomic radius between Au and S results in a brighter Au atom (yellow) and a darker S atom (green) under the same contrast.^[^ ^40^ ^]^ The mapping in Figure S1 (Supporting Information) (Au@S‐rGO) shows that S and Au are dispersed as single atoms on the surface of rGO, further confirming that S and Au exist as single atoms.

**Figure 2:**
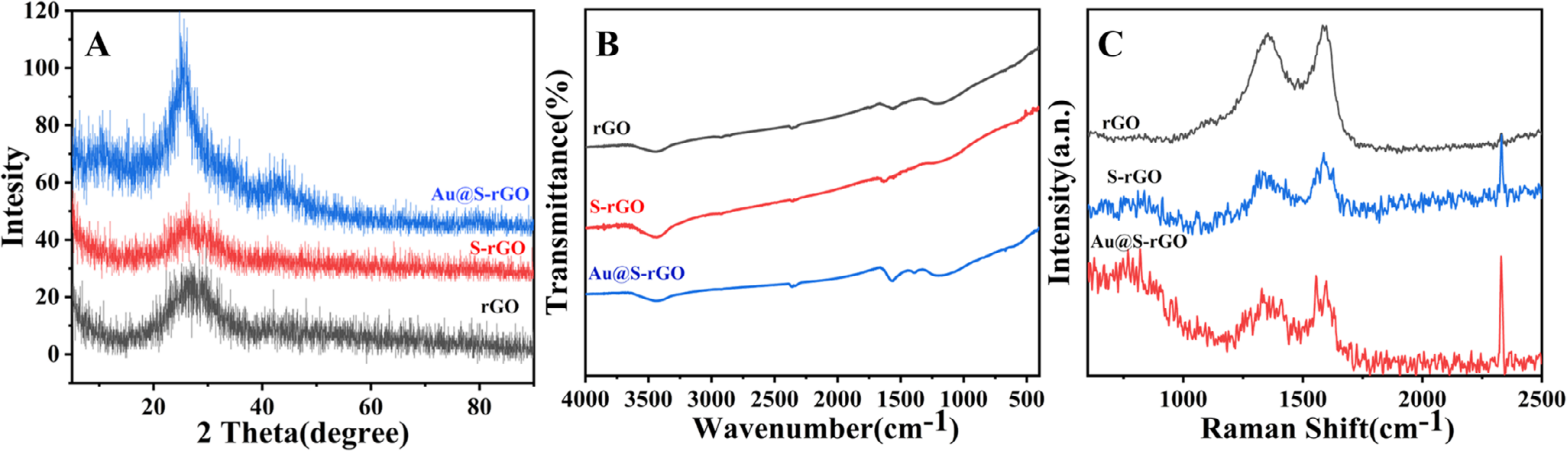
A) XRD, B) FTIR, C) and Raman spectra of rGO, S‐rGO, and Au@S‐rGO.

Further structural characterization of rGO, S‐rGO, and Au@S‐rGO was carried out via X‐ray diffraction (XRD), Fourier transform infrared spectroscopy (FT‐IR), and Raman spectroscopy. As shown in **Figure** **2**A, the crystal structures of the three materials display diffraction peaks at 2theta = 26° and 42°. A comparison of the XRD data reveals that all three peaks are at the same position, confirming that the S and Au on rGO are single atoms that do not affect the crystal structure and corroborate the literature.^[^ ^36^ ^]^

The FT‐IR spectra of rGO, S‐rGO, and Au@S‐rGO obtained by the KBr method in the wavenumber range of 4000–500 cm^−1^ at 25 °C are presented in Figure 2B. No significant differences among the three characteristic peaks are observed, indicating that S and Au are loaded in monad form without forming special functional groups. The Raman spectroscopy data of rGO, S‐rGO, and Au@S‐rGO are compared in Figure 2C. The G‐band of rGO appears at 1596 cm^−1^, whereas the D‐band is visible at 1338 cm^−1^. The peaks of S‐rGO and Au@S‐rGO are no longer apparent, which can be explained by the fact that the S and Au loads cause partial SP^2^ hybridization of the rGO to break down, the atomic layer spacing increases and the other monatom is attached to the rGO through *π*–*π* stacking. This configuration promotes electron transfer in the conjugate complex system, thereby increasing electron mobility.^[^ ^40^ ^]^

### 2.2 Electrochemical Characterization of Sensors

**Figure 3:**
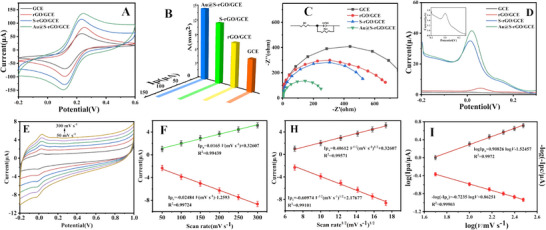
A) CVs, B) electrode active area, C) EIS and D) SWV of the GCE, rGO/GCE, S‐rGO/GCE, and Au@S‐rGO/GCE; E) cyclic voltammetry of the Au@S‐rGO/GCE at different scanning rates; linear plots of F) I versus *V*, H) I versus *V* ^1/2^, I) log I versus log *V*. Statistical data by means ± SD (n = 3).

The electrocatalytic activity of the Au@S‐rGO/GCE was investigated in a solution containing 5.0 mmol L^−1^ [Fe (CN) 6] ^3‐/4−^ and 0.1 mol L^−1^ KCl as the electrolyte via cyclic voltammetry (CV) at a scan rate of 100 mv s^−1^. As depicted in **Figure** **3**A, the electrochemical response of the rGO/GCE and S‐rGO/GCE becomes obvious after rGO and S‐rGO modification, indicating that the material has obvious electrocatalytic activity. However, a further significant increase in peak current and area is observed after the Au@S‐rGO modification. rGO enhances the electronic conductivity and improves the effective area of the electrode, highlighting the synergistic effect of diatomic‐modified materials in promoting the electrochemical response.

Figures S2 (Supporting Information) shows the cyclic voltammetry results of the GCE, rGO/GCE, S‐rGO/GCE, and Au@S‐rGO/GCE at different scanning rates of 5.0 mm [Fe (CN) _6_] ^3‐/4−^ and 0.1 m KCl. The surface area in A (cm^−2^) is then calculated via the Randles–Sevcik equation (Equation (1)), where Ip is the peak current, A represents the effective surface area of the modified electrode, v is the scanning rate of the reaction, and n (n = 1) refers to the corresponding number of transferred electrons. D and C are the diffusion coefficients (D = 7. 6 × 10^−6^ cm^2^ s^−1^) and concentration (C/mol cm^−3^) of potassium ferricyanide solution, respectively.

(1) $$\mathrm{Ip}=2.69\times 10^{5}\times n^{3/2}\times A\times D^{1/2}\times C\times v^{1/2}$$

Figure 3B shows that the electrochemically active region and A values of the bare GCE, rGO/GCE, S‐rGO/GCE, and Au@S‐rGO/GCE are 6.42, 8.88, 12.15, and 14.52 mm^2^, respectively. Accordingly, the Au@S‐rGO/GCE as the modified GCE (14.52 mm^2^) has a significantly greater effective surface area than the bare GCE (6.42 mm^2^). As a result, the Au@S‐rGO composite significantly enhances the active area of the modified GCE, thereby increasing the number of active sites and improving the catalytic activity.

The AC impedance (EIS) diagrams of the different electrodes are displayed in Figure 3C. The greater the radius of the semicircle, the greater the electrode resistance. After fitting the data according to the equivalent circuit diagram, the impedance values of the bare GCE, rGO/GCE, S‐rGO/GCE, and Au@S‐rGO/GCE are 437.9, 291.4, 280.7, and 132.8 Ω, respectively. The impedance of the GCE is the largest, whereas the impedance of the Au@S‐rGO/GCE is the smallest. The conductivity of Au@S‐rGO accelerates electron transfer most effectively.

The square wave voltammetry (SWV) responses of different electrodes to 10 µmol L^−1^ fentanyl in 1/15 mol L^−1^ phosphate buffer solution (PBS) at pH 7.38 are shown in Figure 3D. The oxidation signal of the naked GCE to fentanyl is relatively weak. After rGO modification, the response current of the rGO/GCE to fentanyl was weakly enhanced. After modification with S‐rGO, the oxidation peak current significantly increased. Thus, the single‐atom catalyst S‐rGO has a good catalytic effect on the oxidation of fentanyl. The Au@S‐rGO/GCE further enhances the response signal of fentanyl, which may be due to the synergistic effect of S and Au atoms. The oxidation peak current of fentanyl is ≈1.5‐fold greater than that of S‐rGO/GCE and 76‐fold greater than that of the bare GCE. Consequently, the monatomic catalyst has good application prospects as an efficient electrocatalyst for high‐sensitivity electrochemical sensing platforms.

The electrochemical responses of the Au@S‐rGO/GCE to fentanyl (1.0 µmol L^−1^) at different scan rates are compared in Figure 3E,F. The redox peak current gradually increased with increasing scan rate. In the scanning rate range of 50–300 mV s^−1^, the peak current of fentanyl showed a good linear relationship with the scanning rate (*v*). The linear regression equation is as follows: *Ipa*/µA = 0.0165 *v*/(mV s^−1^) + 0.32607 (R^2^ = 0.99439) and *Ipc*/µA = 0.02484 *v*/(mV s^−1^) − 1.2593 (R^2^ = 0.99724). Therefore, the peak redox current on the Au@S‐rGO/GCE‐modified electrode surface is proportional to the scanning rate, indicating that the electrochemical reaction of fentanyl on the electrode surface is controlled by adsorption. As shown in Figure 3H, the peak current of fentanyl shows a good linear relationship with the square root of the scan rate, indicating that fentanyl oxidation may be a diffusion‐controlled process. In addition, the mapping of log *v* and log I also shows a good linear relationship, with a slope between 0.5 and 1.0 and a slope of 0.8902 (Figure 3I), indicating that the oxidation process of fentanyl is the reaction mainly controlled by adsorption, in which the diffusion of substances also plays an important role in the reaction.

**Figure 4:**
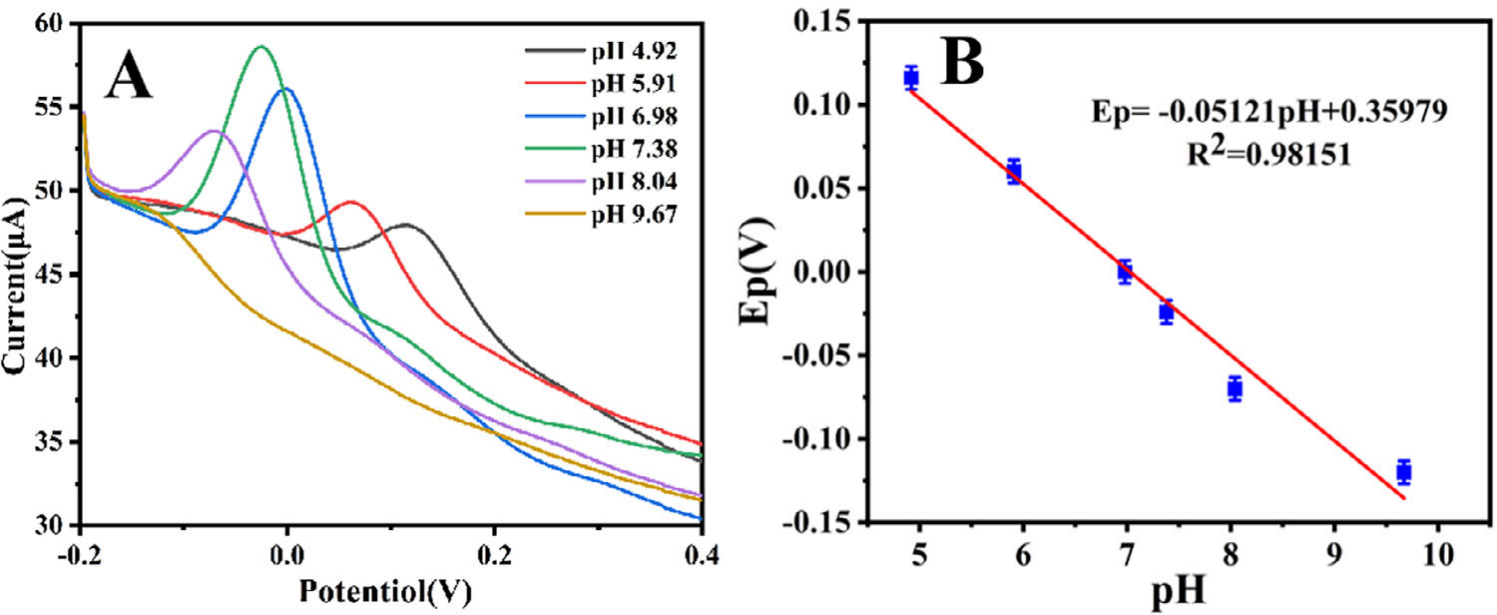
A) SWV response of 1.0 µmol L^−1^ fentanyl at different pH. B) Oxidation potential as a function of pH. Statistical data by means ± SD (n = 3).

The effect of pH on fentanyl oxidation was also studied on the Au@S‐rGO/GCE by determining the response peak current value of a fixed concentration of fentanyl in buffer solutions with different pH values. As shown in **Figure** **4**A, the largest fentanyl response was obtained at a buffer solution pH of 7.38. Fentanyl is a weak base with a pKa of 8.4. At pH 7.38 (close to physiological pH), there is a balance of protonated and neutral forms, and at pH 7.38, fentanyl exists mainly in protonated form and has higher electrochemical activity because it is positively charged and interacts more easily with the electrode surface to promote electron transfer. At lower pH (pH < 7.38): Fentanyl is almost completely protonated, but a high concentration of hydrogen ions in the solution may compete with the electrode surface, resulting in a lower peak current. At higher pH (pH > 7.38): The neutral form predominates, resulting in reduced peak current due to its lack of charge and lower electrochemical activity. At pH 7.38: the balance between the protonated form and the neutral form reaches the best state, which not only ensures the high concentration of deprotonated state, but also avoids the competition of hydrogen ions, so the peak current is maximum. Hence, 7.38 was selected as the best test pH value for subsequent experiments. In addition, the increase in pH resulted in shifts in the oxidation peak potential to the negative potential, indicating that protons participate in the fentanyl oxidation process and proton coupling. As displayed in Figure 4B, the peak potential has a good linear relationship with the pH of the solution, indicating that the reaction involves proton transfer, and the relationship between the potential and pH can be obtained according to Nernst: E = E∘+ n/0.0592 pH. The slope was 0.05121, and n = 1.14 was calculated. Because the number of electrons transferred is an integer, it is 2. Therefore, it is speculated that the reaction of fentanyl on the electrode surface is a process of double electron transfer (Scheme 1), consistent with the reported oxidation process of fentanyl on the electrode surface.^[^ ^21^ ^]^

**Figure 5:**
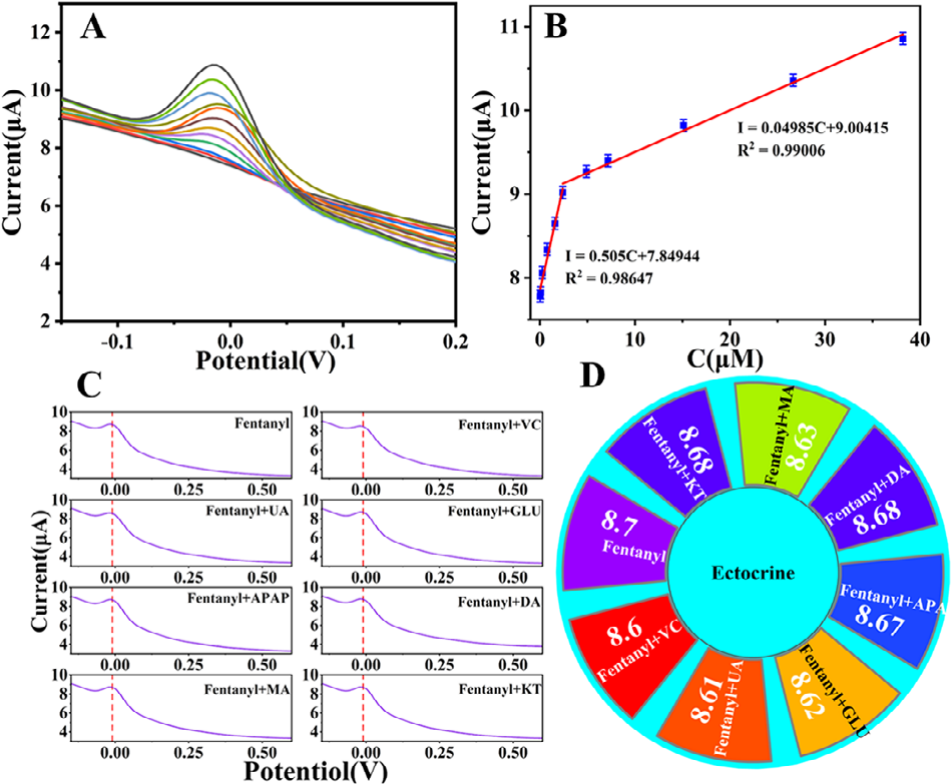
A) SWV of the Au@S‐rGO/GCE for different concentrations of fentanyl. B) Calibration curve of fentanyl. C,D) Influence of different interferents on Au@S‐rGO/GCE detection of 1.0 µmol L^−1^ fentanyl and 50 µmol L^−1^ DA, VC, GLU, UA, APAP, MA, and KT. Statistical data by means ± SD (n = 3).

Under optimal experimental conditions, the prepared Au@S‐rGO/GCE was used to detect different concentrations of fentanyl via the square wave voltammetry technique. As shown in **Figure** **5**A, the peak current value of SWV increased with increasing fentanyl concentration. A good linear correlation is obtained at fentanyl concentrations ranging from 0.0291 to 0.759 µmol L^−1^ and 0.759 to 38.2 µmol L^−1^, laying a reliable foundation for determining the electrochemical fingerprint region of fentanyl. The linear regression equations are *I* = 0.505 C (µmol L^−1^) + 7.84944 (R^2^ = 0.98647) and *I* = 0.04985 C (µmol L^−1^) + 9.00415 (R^2^ = 0.99006). The detection limit was determined to be 9.7 nmol L^−1^, which is lower than the reported acute lethal intoxication concentration of 100 nmol L^−1^ fentanyl (Figure 5B). Over a wide range of concentrations (3–4 orders of magnitude), linear equations of these two different slopes are common, possibly due to the frequent variations in the contributions of the adsorption and diffusion current components.^[^ ^23^ ^]^ At lower concentrations, the sensor exhibits a more linear response, whereas at higher concentrations, the response flattens out owing to saturation effects or limitations at the electrode's active site. Compared with other reported electrochemical detection methods for fentanyl detection, the proposed electrochemical sensor has a unique ability to directly detect low concentrations of fentanyl (Table S1, Supporting Information) coupled with a wide linear range.

### 2.3 Anti‐Interference Research and Actual Sample Analysis

The specificity of the sensing system was evaluated by measuring interference levels in a 1/15 mol L^−1^ phosphate buffer solution at pH 7.38 containing a fentanyl concentration of 1.0 µmol L^−1^ via conventional addition techniques. Chemicals consisting of dopamine (DA, 50.0 µmol L^−1^), ascorbic acid (VC, 50.0 µmol L^−1^), glucose (GLU, 50.0 µmol L^−1^), uric acid (UA, 50.0 µmol L^−1^), acetaminophen (APAP, 50.0 µmol L^−1^), methamphetamine (MA, 50.0 µmol L^−1^), and ketamine (KT, 50.0 µmol L^−1^) are selected as interfering substances. As shown in Figure 5C,D, the response of the Au@S‐rGO/GCE to fentanyl displays relatively weak current changes despite the presence of interfering substances. Therefore, these substances do not interfere with fentanyl detection, demonstrating the good selectivity of the proposed sensor.

The repeatability of the method was tested by manufacturing three identical Au@S‐rGO/GCE electrochemical sensors under the same conditions, followed by an evaluation of their sensitivities to fentanyl detection. At a fentanyl concentration of 1.0 µmol L‐1, the sensors showed a relative deviation of 3.1% in the SWV measurements (Figure S3A–D, Supporting Information). In a parallel detection experiment, the same sensor used for 10 consecutive cycles resulted in a relative deviation of 3.8% (Figure S3B–E, Supporting Information), with a decline in the current response of ≈7.8% after 20 cycles of daily use (two measurements per day for 10 consecutive days) (Figure S3C–F, Supporting Information). Therefore, the proposed sensor shows high reproducibility and stability toward fentanyl detection.

The applicability of the Au@S‐rGO/GCE sensor in real samples was evaluated by testing fentanyl in serum, and the recovery rate was determined via the standard addition method. As depicted in **Table** **1**, recovery rates ranging from 90.0% to 104% are obtained, with a reproducibility (expressed as relative standard deviation, RSD) of less than 3.2%, indicating the reliability of the sensor for fentanyl detection.

**Table 1:** Determination of fentanyl in serum samples using Au@S‐rGO/GCE (n = 3).

Sample	Added/µm	Detected/µm	Recovey [%]	RSD [%]
Serum	0.05	0.052	104	3.2
	0.1	0.09	90	2.4
	1	0.99	99	1.8
	10	0.98	98	1.6

The feasibility of rapid detection of fentanyl by sensors was explored through the detection of blood samples from rats simulating drug users (**Table** **2**). Within 240 min, the detection data of the Au@S‐rGO/GCE were highly consistent with those of liquid chromatography tandem mass spectrometer (LC‐MS/MS), and the consistency was greater than 85.7%. In view of the existence of human error and systematic error in actual detection, this deviation is within the standard range that meets the actual detection requirements. The sensor also showed good performance in the detection of real blood samples from untreated rats. In summary, the developed Au@S‐rGO/GCE has broad application prospects for actual field detection.

**Table 2:** Au@S‐rGO/GCE compared with LC‐MS/MS for detecting fentanyl in blood samples from actual rats.

Sample	Time min^−1^	LC‐MS/MS [µm]	Au@S‐rGO/ GCE [µm]	Au@S‐rGO‐GCE/ LC‐MS/MS [%]
Rat serum	5	0.6271	0.58	92.5
	15	0.5736	0.52	90.7
	30	0.4963	0.43	89.1
	60	0.4041	0.36	89.1
	120	1.0788	1.01	93.6
	240	0.0713	0.06	85.7

### 2.4 Mechanism Analysis

**Figure 6:**
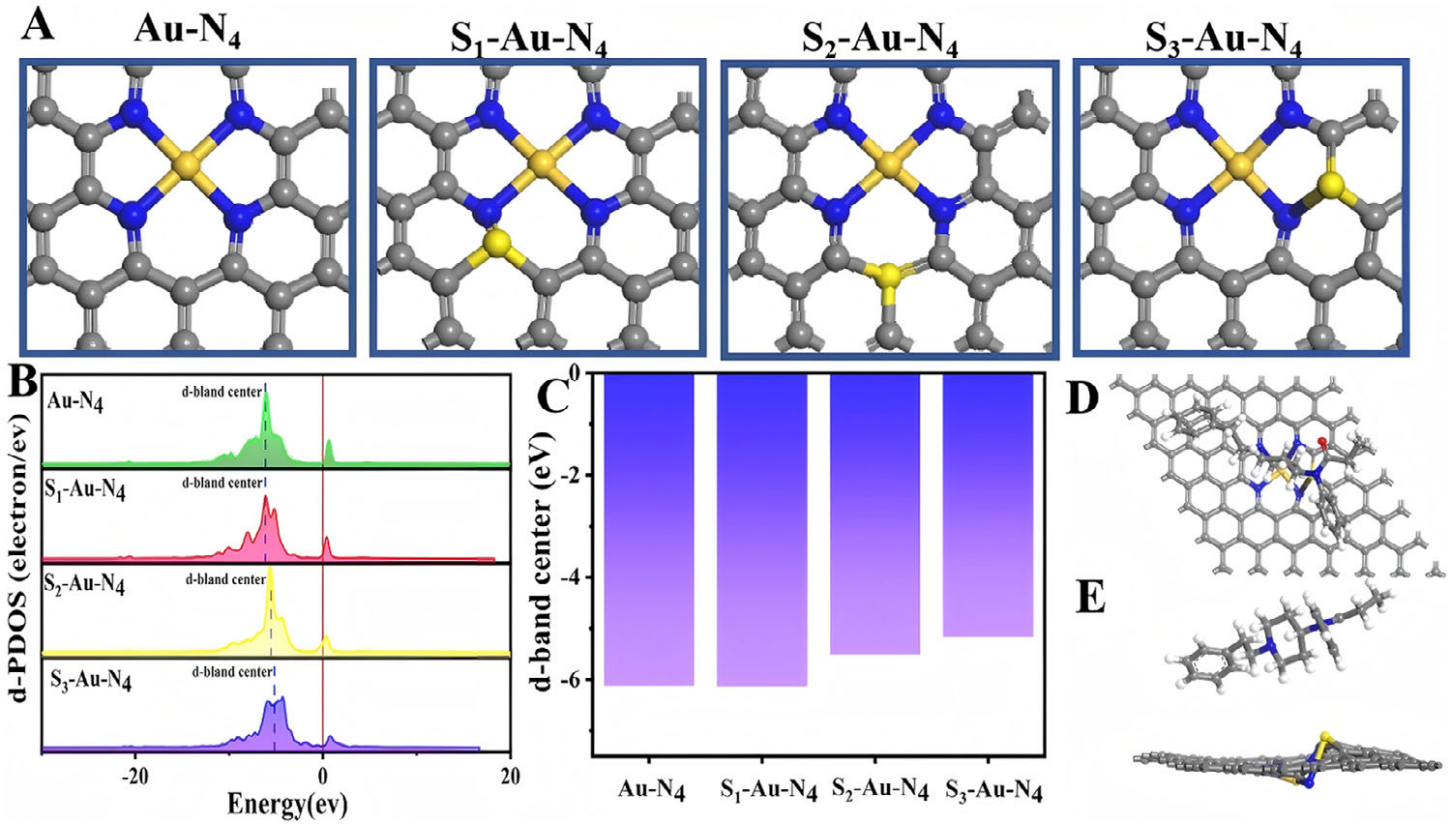
A) Top view of the atomic structure of different S‐position Au‐N_4_: S_1_‐Au‐N_4_, S_2_‐Au‐N_4_, and S_3_‐Au‐N_4_. The skewness density (PDOS) of the Au‐d band B) and the *d*‐band center C) at different local locations in S‐doped Au‐N_4_ are compared with those in Au‐N_4_. Fentanyl adsorption configuration on the surface of Au@S‐rGO: D) top view and E) side view.

Three possible locations of S‐doping at the periphery of the Au‐N_4_ segment are simulated via density functional theory (DFT) calculations, and the differences in the electronic structures at different S locations are determined via computation. **Figure** **6**A shows the three S positions of the Au‐N_4_ section, named S_1_‐Au‐N_4_, S_2_‐Au‐N_4_, and S_3_‐Au‐N_4_. The projected partial density of states (PDOS) of the partial Au atoms S_1_‐Au‐N_4_, S_2_‐Au‐N_4_, and S_3_‐Au‐N_4_ are presented in Figure 6B. The PDOS studies of the Au atoms revealed a much narrower D‐band gap for the atoms in S_x(1, 2, 3)_‐Au‐N4 than for the other Au‐N_4_ atoms, with S_3_‐Au‐N_4_ displaying the narrowest D‐band gap, which can be explained by the improvement in the local electronic conductivity of the Au‐N_4_ segment by adjacent S‐doping. In addition, the D‐band center is highly correlated with the adsorption of molecules on metal surfaces and serves as an effective descriptor of the catalytic activity of transition metal catalysts. In Figure 6C, the nonmetallic element S is introduced to regulate the electronic structure of the catalytic active center, and the *d*‐band center of the Au atom experiences varying degrees of elevation with the specific c site of sulfur doping. When the center of the D band is near the Fermi level, the electronic structure of the metal surface is aligned with the energy of the reactants, and electron exchange with the adsorbent is more effective, thus increasing the adsorption strength. This phenomenon usually manifests as enhanced catalytic activity. Moreover, metals with D band centers close to the Fermi level can provide appropriate adsorption energy in the catalytic reaction, which is very important for the adsorption, activation, and conversion of reactants in the catalytic process. Especially in the catalytic process, optimizing the adsorption energy can reduce the energy barrier of the reaction and increase the reaction rate. By adjusting the position of the D band center, the catalyst can achieve stronger adsorption performance and higher catalytic activity. The S_3_‐Au‐N_4_ configuration has the highest *d*‐band center, reaching −5.157 eV, closest to the Fermi level and exhibiting the best adsorption effect.^[^ ^36^ ^]^

The charge density in different configurations is depicted in Figure S4A (Supporting Information). The Au atoms are surrounded by red regions, whereas the N atoms are surrounded by yellow regions, indicating that the Au atoms have a higher charge density and stronger chemical activity. The differential charge density diagram of the four configurations in Figure S4B (Supporting Information) reveals an Au atom surrounded by blue, suggesting the transfer of electrons to the surrounding N atom, which can be further supported by the presence of N atoms surrounded by red in the S_3_‐Au‐N_4_ configuration.

Further analysis of the Mulliken charges for the four configurations (Figure S5, Supporting Information) revealed a greater charge of the Au atom in the S_3_‐Au‐N_4_ configuration before and after doping with the S atom, indicating an obvious reduction. Moreover, the S atom in the S_3_‐Au‐N_4_ configuration has the lowest charge of 0.330e, indicating a loss of electrons to the N atom simultaneously by both the Au atom and the S atom. Consequently, the doping of nonmetallic atoms is expected to regulate the electronic structure of the catalytically active center to increase the catalytic activity of single‐atom catalysts. Moreover, through experimental observation, Figure 1H shows that the spacing between S atoms and Au atoms is 3.208 Å, which is exactly consistent with the atomic spacing between S and Au in the S3‐Au‐N4 model (Figure S6, Supporting Information). The simultaneous insertion of S and Au causes fluctuations in the graphene atomic plane, thus affecting the SP^2^ stacking between graphene layers, which is consistent with the Raman characterization in Figure 2C. Accordingly, the S_3_‐Au‐N_4_ model is selected for subsequent calculations.

Moreover, the electrostatic potential of fentanyl in Figure S7 (Supporting Information) shows that the hydrogen on the C atom connected to piperidine nitrogen is red, indicating that it lacks electrons and reacts more easily with charged substances. Additionally, Tables S2 and S3 (Supporting Information) present the Fukui functions of fentanyl and Au@S‐rGO, respectively, and their electrophilic attack indices, nucleophilic attack indices, and free radical indices were analyzed. Figures S8 and S9 (Supporting Information) present the individual atoms of fentanyl and Au@S‐rGO, respectively. The H42 on the fentanyl piperidine ring has the highest electrophilic attack index of 0.12, making it susceptible to electronic attack. H34 also has a high electrophilic attack index and, like H42, is also an H on the C atom linked to the piperidine nitrogen. Two hydrogen atoms are easily attacked by electrons to form proton H departions. It is speculated that the oxidation process of fentanyl on the electrode surface is the process of double proton H leaving after being attacked by double electrons and then forming nofentanyl and phenacetaldehyde, consistent with the reported oxidation process of fentanyl on the electrode surface.^[^ ^21^ ^]^ Moreover, the Au atom on graphene has the highest nucleophilic attack index, rendering it vulnerable to nucleophilic attack. Consequently, the H on the piperidine ring and the Au on the Au@S‐rGO were selected as the interaction sites for the construction of the adsorption model.

To further explore the active center of interaction between the Au@S‐rGO monatomic material and fentanyl and the changes in the electronic structure of the material after the interaction, we constructed and optimized the adsorption configuration of fentanyl on Au@S‐rGO and the most stable adsorption configuration of fentanyl is shown in Figure 6D,E. The adsorption energy of fentanyl on Au@S‐rGO was studied with Dmol3. All the DFT calculations are performed via the Becke‐Lee‐Yang‐Parr functionals within the generalized gradient approximation (GGA). Spin polarization was also considered in the calculation.

The adsorption energy was calculated via the following formula: ∆E = E_Au@S‐rGO‐fentanyl –_ (E _Au@S‐rGO_ + E _fentanyl_). The adsorption energy is calculated as −30.43 kcal mol^−1^ (Table S4, Supporting Information), indicating that fentanyl tends to spontaneously adsorb on the Au@S‐rGO surface, which is conducive to the sensitive detection of fentanyl.

## 3 Conclusion

In summary, an electrochemical sensor for fentanyl detection was constructed by obtaining atomically dispersed gold‐ and sulfur‐modified reduced graphene oxide via the solvothermal method. The simulation results showed that the doping of S improved the electronic structure of the Au element and significantly improved the catalytic activity of the electrodes for fentanyl detection. The prepared detection platform exhibited a good linear relationship and a low detection limit. The detection of fentanyl in human serum samples indicated recovery rates between 98.0% and 104%. The feasibility of fentanyl detection in actual use was further verified in blood samples from actual rats. After injection of the drugs, fentanyl levels in blood samples in the first 240 min are highly consistent with those obtained by mass spectrometry. Overall, the Au@S‐rGO diatomic catalyst design looks promising for the construction of excellent electrochemical sensing platforms for the practical detection of fentanyl and can be expanded to other drugs.

## 4 Experimental Section

The chemicals and apparatus used are described in the Supporting Information. All animal operations were conducted in compliance with Kunming Medical University's rules for animal experimentation.

### Preparation of S‐rGO

Sulfur atom‐modified rGO nanosheets were synthesized via a hydrothermal method. Thioacetamide acts as both an N source and an S source. Initially, 0.05 g of reduced graphene oxide (rGO) was dispersed in a 25 mL isopropanol solution, and the suspension was sonicated for 30 min. Once homogenized, thioacetamide with a sulfur‐containing element‐to‐rGO mass ratio of 0.15% was added to the rGO dispersion under magnetic stirring. The suspension was treated under high‐speed stirring for 2 h, after which the solution was transferred to an autoclave and allowed to react at 180 °C for 24 h. Upon termination of the reaction, the suspension was sequentially washed with ultrapure water and ethanol. Finally, the product was filtered and dried under vacuum at 60 °C for 10 h to obtain single‐atom sulfur‐modified reduced graphene oxide nanosheets (S‐rGO).

### Preparation of Au@S‐rGO

Synthesis of gold‐ and sulfur diatomically modified rGO nanosheets by a hydrothermal method. First, 0.03 g of S‐rGO was dispersed in 25 mL of ultrapure water via ultrasonication for 30 min. Following homogenization, a gold trichloride solution with a 0.15% mass ratio of gold to S‐rGO was added to the rGO dispersion under continuous magnetic stirring. After adding 5 mL of 14.55 mol L^−1^ sodium citrate solution, the resulting mixture was agitated at high speed for 2 h. The solution was subsequently transferred to an autoclave and reacted at 100 °C for 6 h. Upon reaction completion, the resulting suspension was repeatedly washed with ultrapure water and ethanol. Finally, the product was filtered and dried under vacuum at 60 °C for 10 h to obtain gold‐ and sulfur diatomically modified reduced graphene oxide (Au@S‐rGO) nanosheets.

### Preparation of S‐rGO/GCE and Au@S‐rGO/GCE

First, 1 mg of S‐rGO powder was dispersed in 1 mL of ultrapure water and ultrasonically mixed for 30 min to form a uniform suspension. Then, the S‐rGO‐modified glass carbon electrode (S‐rGO/GCE) was obtained by applying 7 µL suspension droplets on the surface of the treated glass carbon electrode and treating it with infrared light for 10 min. The preparation method of the Au@S‐rGO‐modified glassy carbon electrode (Au@S‐rGO/GCE) was similar.

### Preparation of Human Serum Samples

Serum samples were collected from healthy volunteers and stored in a refrigerator (4 °C) overnight. A volume of ≈3 mL of serum was withdrawn, after which 30 µL of perchloric acid (HClO_4_, 20%/vol) was added. The sample was subsequently stirred vigorously for 2 min and centrifuged at 6000 rpm for 10 min to obtain the precipitated protein. After centrifugation, the samples were diluted with PBS (1/15 mol L^−1^ phosphate buffer solution, pH 7.38) for electrochemical analysis. The standard addition method was used to determine fentanyl levels in serum samples.

### Preparation of Actual Blood Samples from Rats

Healthy male rats (weighing 200–250 g) were first selected and then kept in a clean, quiet environment for at least one week. To reduce the effect of food on drug absorption, the rats were allowed to fast for 12 h, and a 1 mg mL^−1^ fentanyl solution (0.3–0.375 mL) was injected intraperitoneally. Under deep anesthesia at the set time points (5, 15, 30, 60, 120, and 360 min), abdominal aortic blood was collected in the anticoagulant tube (three rats were collected at each time point). Afterward, 10 µL of the internal standard SKF525A (1 µg mL^−1^) was added to 1 mL of blood, followed by the addition of 2 mL of boric acid buffer (pH 9.2). Next, 4 mL of ether was extracted, spun to mix, and centrifuged. The supernatant was then removed, and the blood was dried in a water bath at 60 °C. The obtained residue was redissolved with 200 µL of mobile phase (A:B = 30:70) and a 0.22 µm organic microporous filter membrane for analysis and testing.

### Computational Models and Mechanisms

All three Au@S‐rGO models were constructed with the Material Studio 2020 software package, which is based on a cyclic 4 × 4 graphene monolayer of a carbon framework. Two carbon atoms were subsequently removed, and then four carbon atoms around the dispersion site were replaced with four nitrogen atoms, providing an anchoring site for the gold atoms. The carbon atoms close to the center Au‐N₄ were partially replaced by sulfur atoms to explore the possible Au@S‐rGO structure. Three existing S‐atom locations near the center of Au‐N₄ were constructed and calculated them via density functional theory (DFT) in CASTEP. All density functional theory (DFT) calculations were performed via the GGA via the Perdew–Burke–Ernzerhof functionals. In a 16 Å vacuum space, the lattice was optimized via a 2 × 2 × 1 Monkhorst – Pack type K‐point sampling and a periodic – plate model. The cutoff energy was set to 571 eV, with a convergence standard of an energy change of 1 × 10⁻⁶ eV. Spin polarization was also considered in the calculation.

## Conflict of Interest

The authors declare no conflict of interest.

## Supporting information



Supporting Information

## Data Availability

The data that support the findings of this study are available from the corresponding author upon reasonable request.
