# Supplementary material for: Dual‐Atom Catalyst Au@S‐rGO for Rapid and Highly Sensitive Electrochemical Detection of Fentanyl in Serum
Source: Adv Sci (Weinh). 2025 Mar 16;12(18):2500430. doi: 10.1002/advs.202500430 (PMC12079473; doi:10.1002/advs.202500430)
Supplement: Supplementary file 1 — Supporting Information [file ADVS-12-2500430-s001.docx]

Dual-Atom Catalyst Au@S-rGO for Rapid and Highly Sensitive Electrochemical Detection of Fentanyl in Serum

Meng Li^b,#^, ZhiJin Fan^a,#^, Qiuxia Gao^a,#^, Ying He^b^, Anyun Xu^b^, Zhaofeng Gu^b^, Shixiong Wang^b^, Huiping Bai^b,^*, Yuhui Liao^a,^*, Ruilin Zhang^a,^*

^a^School of Forensic Medicine, NHC Key Laboratory of Drug Addiction Medicine, Institute for Engineering Medicine, Kunming Medical University, Kunming, 650500, Yunnan, China.

^b^School of Material and Energy; Institute of International Rivers and Eco-Security; School of Chemical Science and Technology; Yunnan University, Kunming 650091, China.

*Corresponding Authors: zrlorg@126.com (R. Zhang); liaoyh8@mail.sysu.edu.cn (Y. Liao); baihuiping@ynu.edu.cn (H. Bai)

#These authors contributed equally to this work.

1. **Materials and methods**

*1.1 Chemicals and apparatus*

Fentanyl (FEN), methamphetamine (MA), and ketamine (KT) were provided by Kunming Medical University (Kunming, China). Potassium ferricyanide, disodium hydrogen phosphate, sodium citrate, potassium dihydrogen phosphate, isopropyl alcohol, methanol and acetic acid were purchased from Fengchuan Chemical Reagents Co., LTD. (Tianjin, China). Uric acid (UA), Vitamin C (VC), glucose (GLU) and acetaminophen (APAP) were obtained from Sinopharm Chemical Reagent Co., LTD (Shanghai, China). Reduced graphene oxide (rGO) was supplied by Xianfeng Nano (Nanjing, China). Gold trichloride solution and thioacetamide were purchased from Yuanye Biotechnology Co., LTD (Shanghai, China).

All experiments were conducted using a CHI660E electrochemical workstation (Shanghai CH Instruments Co., China). Synthesized nanomaterials were characterized by the Hitachi S520 field-emission scanning electron microscope (SEM, Tokey), TTRIII transtarget X-ray diffractometer (XRD, Tokey), JEM-2100 transmission electron microscope (TEM, Japan), Titan themes G2300 High Angle annular dark field aberration electron microscope (STEM, ThermoFisher), 640-IR Fourier infrared spectrometer (FIR, Shanghai Lyrey), RenishawinVia Confocal microscopic Raman spectrometer (Raman, Renishaw) and LCMS-8040 triple quadrupole liquid chromatography tandem mass spectrometer (LC-MS/MS, Shimadzu).

**
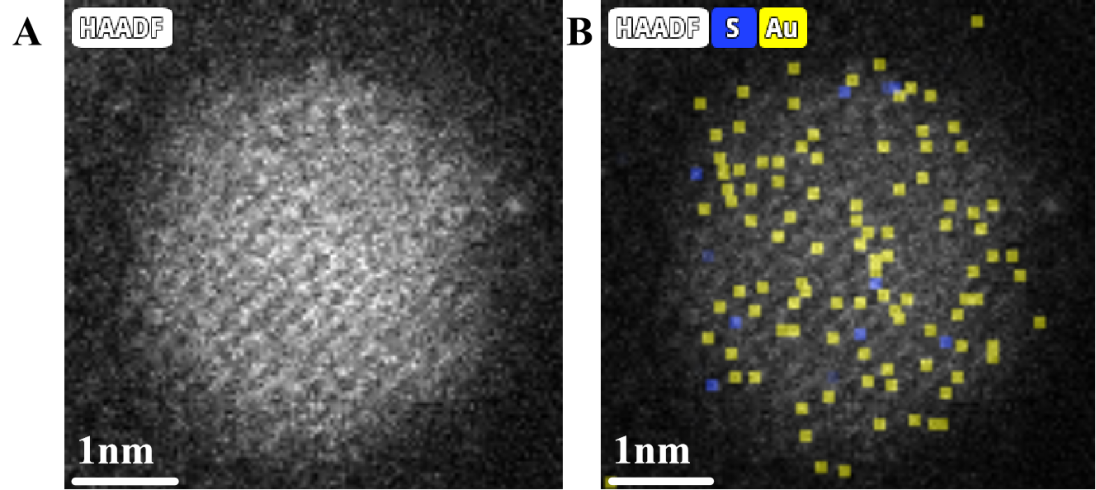
**

**Figure S1.** (A) STEM images and (B) elemental mapping of Au@S-rGO.

**
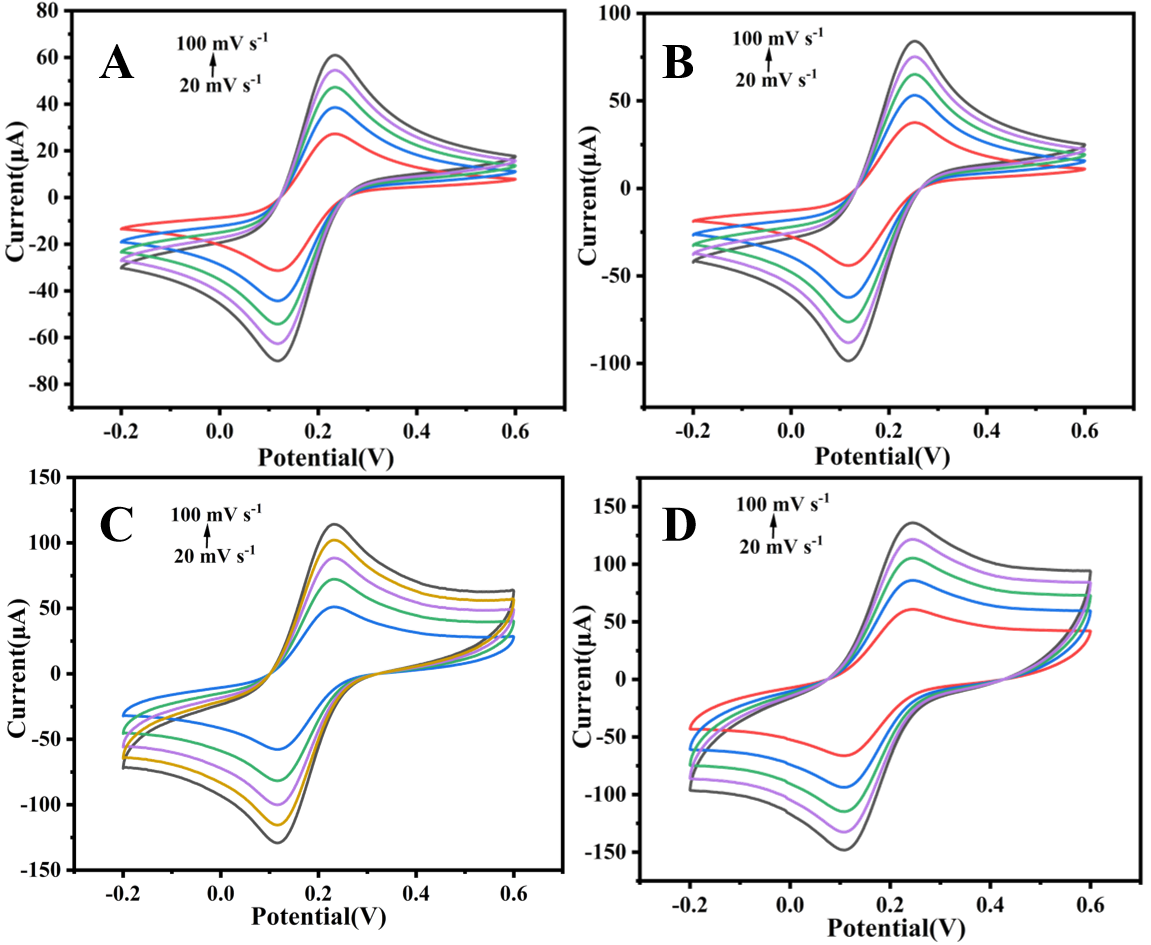
**

**Figure S2.** Cyclic voltammetry of GCE, rGO/GCE, S-rGO/GCE and Au@S-rGO/GCE at different scanning rates of 5.0 mmol L^-1^ [Fe (CN) 6] ^3-/4-^ and 0.1 mol L^-1^ KCl .

**Table S1.** Comparison of other electrochemical methods for determination of fentanyl.

| **Electrode** | **Electrode Modification** | **Methods** | **Linear range** | **Limit of detection** | **Ref.** |
| --- | --- | --- | --- | --- | --- |
| **Carbon** | SWCNTs | DPV | 0.01-1 μM | 11 nM | 4 |
| **GCE** | Carbon nanoonions | DPV | 1-60 μM | 0.3 μM | 1 |
| **Carbon SPE** | MWCNTs and ionic liquid | SVW | 10-100 μM | 10 μM | 2 |
| **Carbon SPE** | Zn-MOF | DPV | 1-100 μM | 0.3 μM | 3 |
| **Carbon SPE** | Ionic liquid | SWV | 5-100 μM | 5 μM | 6 |
| **Hollow microneedle** | Carbon paste | SWV | 10-200 μM | 10 μM | 5 |
| **Carbon SPE** | NiMnO_3_/MWCNTs | DPV | 0.05–10 μM | 0.02 μM | 7 |
| **GCE** | COF@rGO | SWV | 0.1-6.5 μM | 33 nM | 9 |
| **GCE** | MIM@ErGO | SWV | 0.0038-1.72 μM | 1.28 nM | 8 |
| **GCE** | Au@S-rGO | SWV | 0.0291-0.759 μM | 9.7 nM | This work |

**
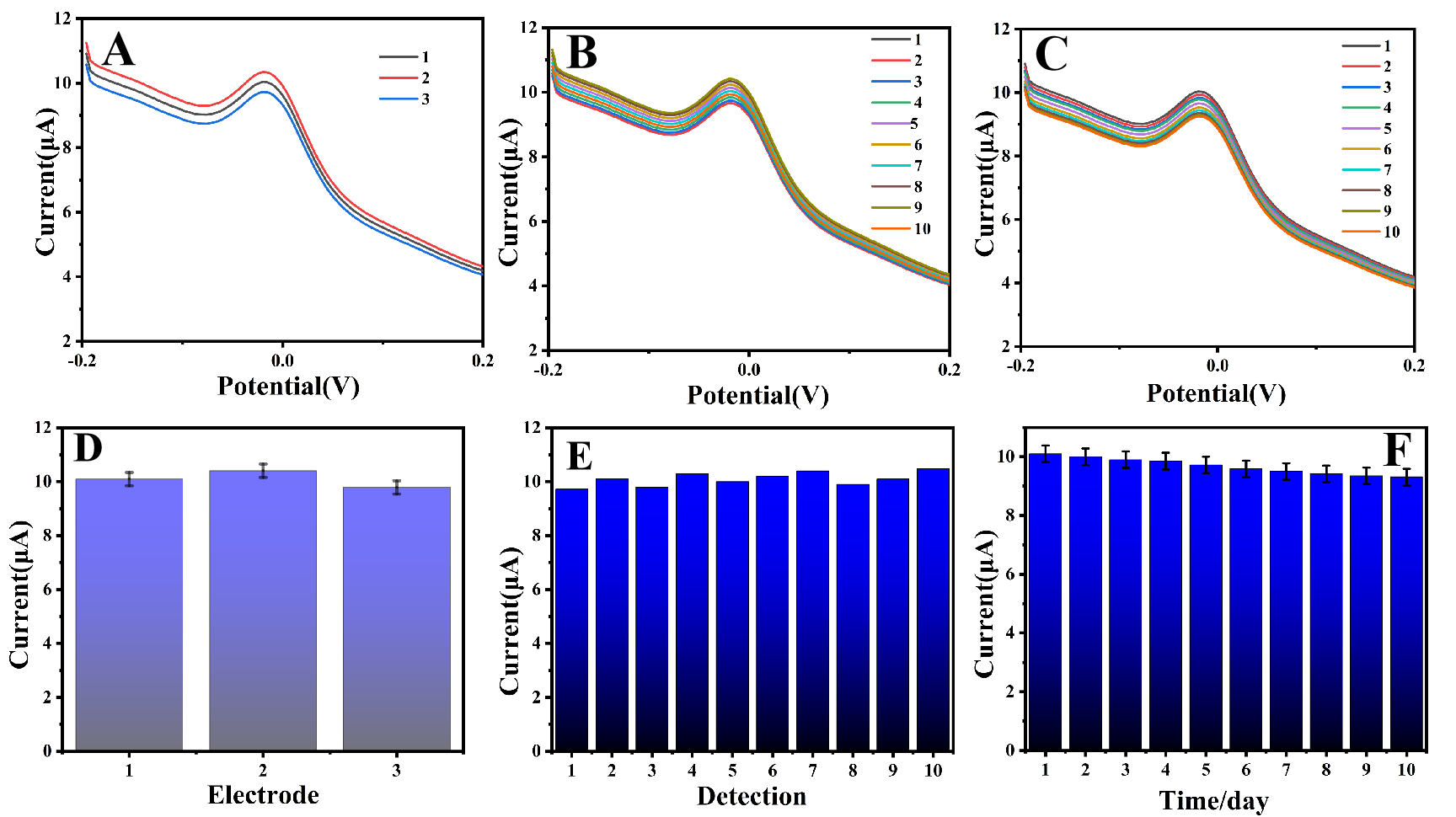
**

**Figure S3.** (A) SWV diagram and (D) the peak current responses of three parallel electrodes to 1 μM fentanyl; (B) SWV diagram and (D) The peak current responses of the same electrode to 1μM fentanyl for 10 consecutive times; (C) The SWV diagram and (F) the current change trend of the same electrode within 10 days.

**
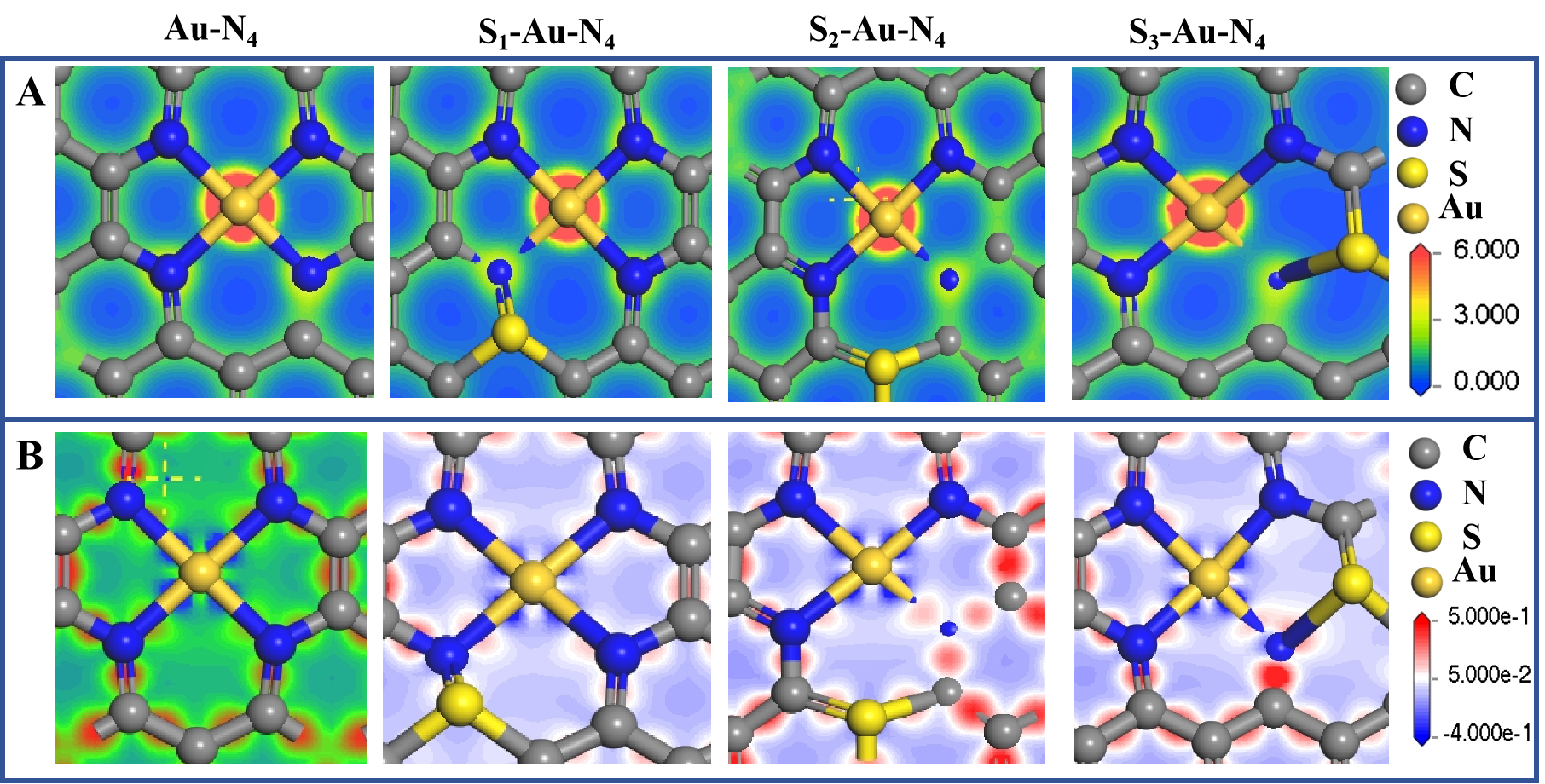
**

**Figure S4.** Charge density diagram (A) and differential charge density diagram (B) of the four structures.

**
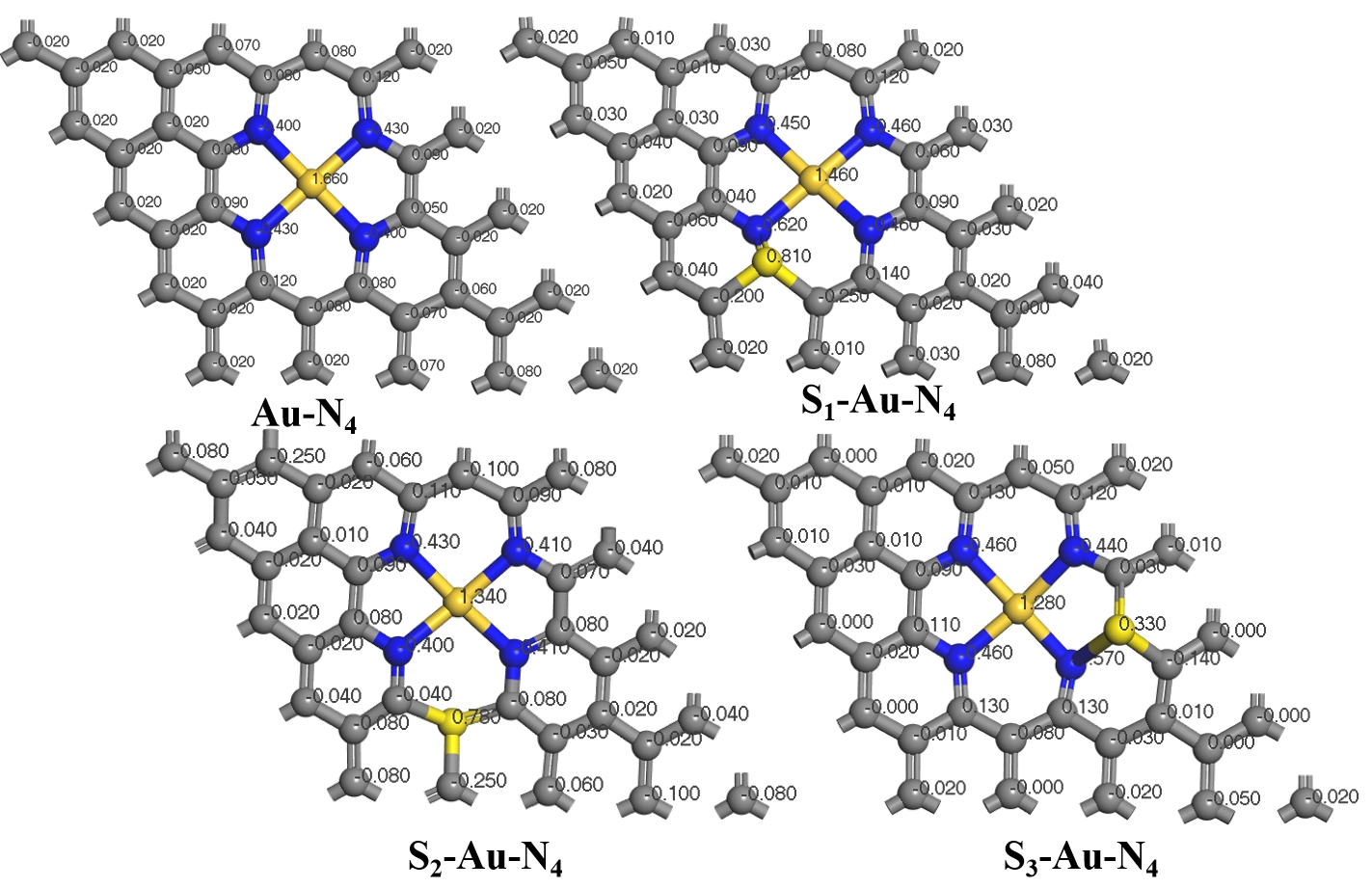
**

**Figure S5.** Mulliken charges for the four structures.


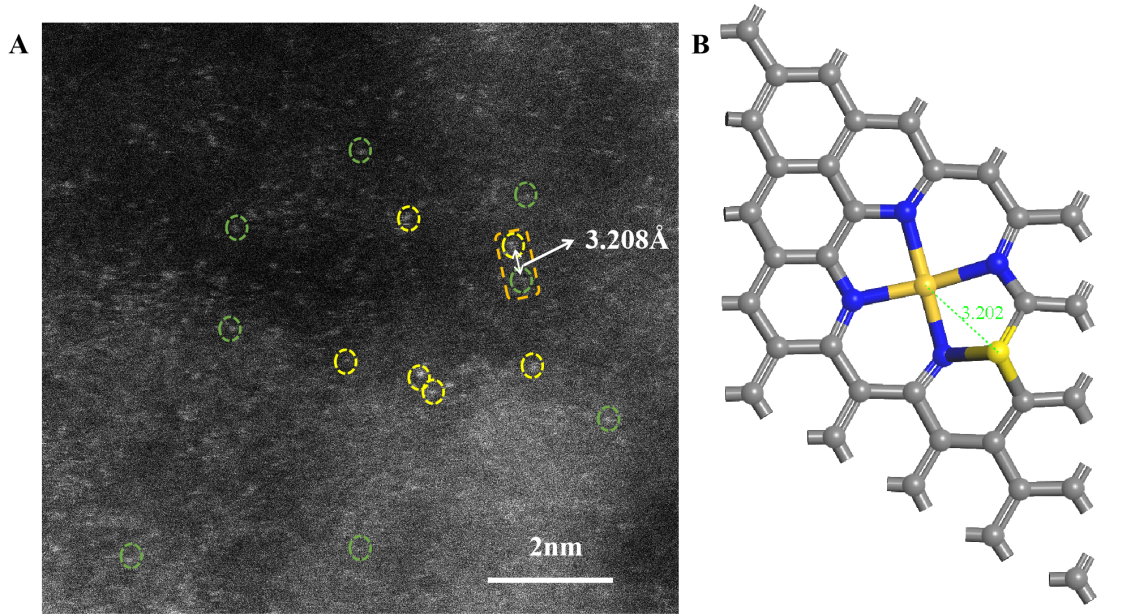


**Figure S6.** STEM of Au@S-rGO and Top view of the atomic structure of S_3_-Au-N_4_


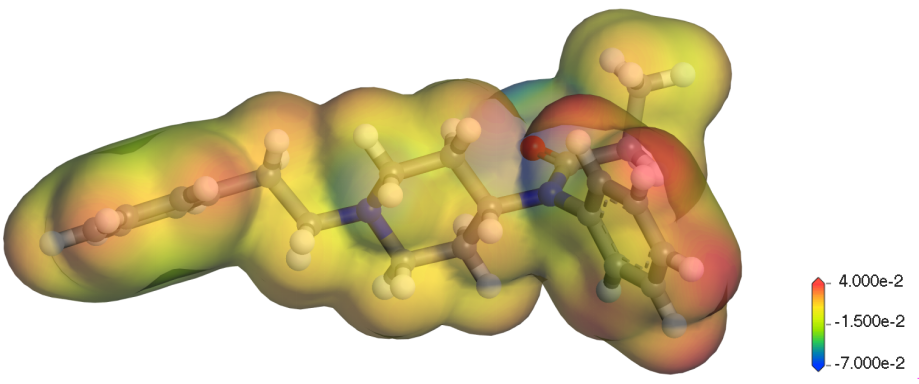


**Figure S7.** Electrostatic potential diagram of fentanyl.

**
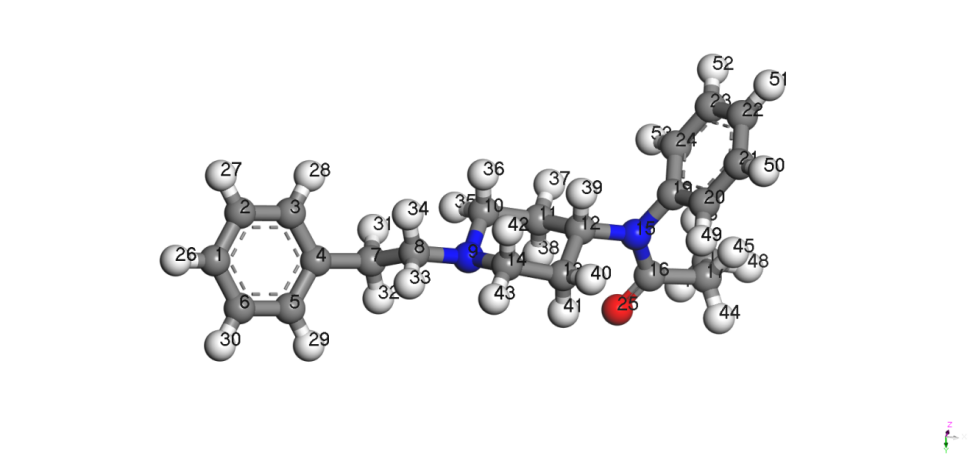
**

**Figure S8.** Fentanyl atomic label.

**Table S2.** Fukui function analysis of fentanyl atoms.

| Atom | Fukui Indices for Electrophilic Attack (Fukui(-)) | Fukui Indices for Nucleophilic Attack (Fukui(+)) | Fukui Indices for Radical Attack (Fukui(0)) |
| --- | --- | --- | --- |
| C(1) | 0.011 | 0.003 | 0.007 |
| C(2) | 0.007 | 0.002 | 0.004 |
| C(3) | -0.005 | -0.001 | -0.003 |
| C(4) | -0.016 | -0.004 | -0.01 |
| C(5) | 0.001 | -0.001 | 0.000 |
| C(6) | 0.006 | 0.002 | 0.004 |
| C(7) | -0.024 | -0.003 | -0.014 |
| C(8) | -0.053 | -0.012 | -0.032 |
| N(9) | 0.198 | 0.000 | 0.099 |
| C(10) | -0.056 | 0.001 | -0.027 |
| C(11) | -0.023 | -0.006 | -0.014 |
| C(12) | -0.006 | -0.017 | -0.011 |
| C(13) | -0.023 | -0.005 | -0.014 |
| C(14) | -0.055 | 0.000 | -0.028 |
| N(15) | -0.002 | -0.013 | -0.007 |
| C(16) | 0.009 | 0.016 | 0.013 |
| C(17) | -0.007 | -0.018 | -0.013 |
| C(18) | -0.007 | -0.007 | -0.007 |
| C(19) | -0.013 | 0.013 | 0.000 |
| C(20) | -0.006 | 0.055 | 0.025 |
| C(21) | 0.004 | 0.114 | 0.059 |
| C(22) | 0.007 | 0.008 | 0.007 |
| C(23) | 0.004 | 0.053 | 0.028 |
| C(24) | -0.006 | 0.127 | 0.061 |
| O(25) | 0.025 | 0.054 | 0.039 |
| H(26) | 0.032 | 0.012 | 0.022 |
| H(27) | 0.027 | 0.008 | 0.018 |
| H(28) | 0.003 | -0.004 | 0.000 |
| H(29) | 0.011 | 0.001 | 0.006 |
| H(30) | 0.029 | 0.010 | 0.019 |
| H(31) | 0.029 | 0.001 | 0.015 |
| H(32) | 0.033 | 0.004 | 0.018 |
| H(33) | 0.058 | 0.011 | 0.035 |
| H(34) | 0.108 | 0.010 | 0.059 |
| H(35) | 0.065 | 0.020 | 0.043 |
| H(36) | 0.119 | 0.003 | 0.061 |
| H(37) | 0.053 | -0.004 | 0.025 |
| H(38) | 0.028 | 0.018 | 0.023 |
| H(39) | 0.02 | -0.007 | 0.006 |
| H(40) | 0.054 | 0.000 | 0.027 |
| H(41) | 0.028 | 0.018 | 0.023 |
| H(42) | 0.12 | 0.004 | 0.062 |
| H(43) | 0.066 | 0.021 | 0.044 |
| H(44) | 0.02 | 0.040 | 0.030 |
| H(45) | 0.02 | -0.025 | -0.002 |
| H(46) | 0.006 | -0.005 | 0.000 |
| H(47) | 0.007 | 0.032 | 0.019 |
| H(48) | 0.025 | 0.031 | 0.028 |
| H(49) | 0.003 | 0.082 | 0.043 |
| H(50) | 0.021 | 0.095 | 0.058 |
| H(51) | 0.024 | 0.085 | 0.055 |
| H(52) | 0.02 | 0.089 | 0.054 |
| H(53) | 0 | 0.087 | 0.044 |

**
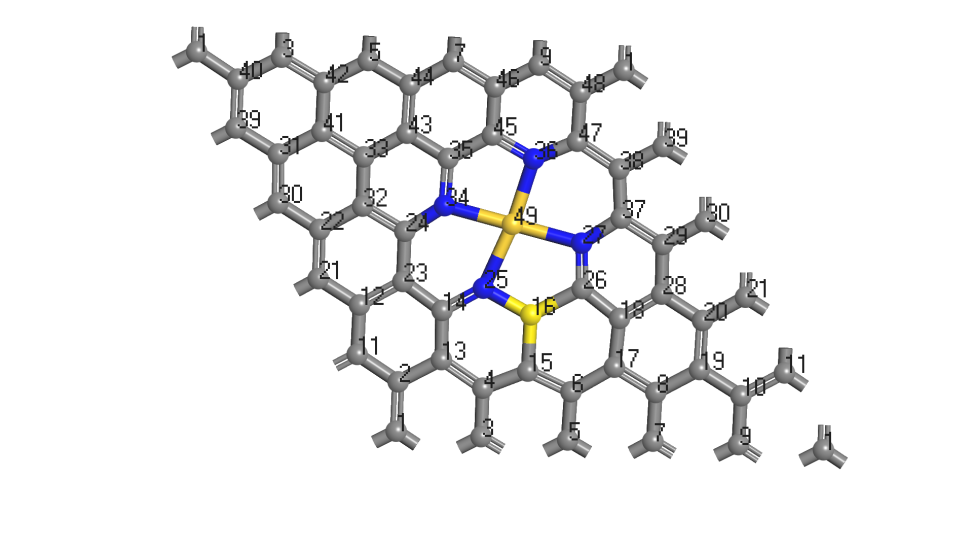
**

**Figure S9.** Au@S-rGO atomic label.

**Table S3.** Fukui function analysis of Au@S-rGO atoms.

| Atom | Fukui Indices for Electrophilic Attack (Fukui(-)) | Fukui Indices for Nucleophilic Attack (Fukui(+)) | Fukui Indices for Radical Attack (Fukui(0)) |
| --- | --- | --- | --- |
| C(1) | 0.02 | 0.02 | 0.02 |
| C(2) | 0.016 | 0.016 | 0.016 |
| C(3) | 0.015 | 0.015 | 0.015 |
| C(4) | 0.023 | 0.023 | 0.023 |
| C(5) | 0.015 | 0.015 | 0.015 |
| C(6) | 0.02 | 0.02 | 0.02 |
| C(7) | 0.022 | 0.021 | 0.022 |
| C(8) | 0.014 | 0.014 | 0.014 |
| C(9) | 0.025 | 0.025 | 0.025 |
| C(10) | 0.015 | 0.015 | 0.015 |
| C(11) | 0.021 | 0.021 | 0.021 |
| C(12) | 0.016 | 0.017 | 0.017 |
| C(13) | 0.022 | 0.021 | 0.022 |
| C(14) | 0.029 | 0.029 | 0.029 |
| C(15) | 0.016 | 0.016 | 0.016 |
| S(16) | 0.052 | 0.052 | 0.052 |
| C(17) | 0.025 | 0.025 | 0.025 |
| C(18) | 0.019 | 0.019 | 0.019 |
| C(19) | 0.021 | 0.021 | 0.021 |
| C(20) | 0.017 | 0.017 | 0.017 |
| C(21) | 0.02 | 0.019 | 0.02 |
| C(22) | 0.02 | 0.02 | 0.02 |
| C(23) | 0.016 | 0.016 | 0.016 |
| C(24) | 0.031 | 0.031 | 0.031 |
| N(25) | 0.001 | 0.001 | 0.001 |
| C(26) | 0.021 | 0.022 | 0.022 |
| N(27) | 0 | 0 | 0 |
| C(28) | 0.019 | 0.019 | 0.019 |
| C(29) | 0.02 | 0.02 | 0.02 |
| C(30) | 0.016 | 0.017 | 0.017 |
| C(31) | 0.024 | 0.024 | 0.024 |
| C(32) | 0.017 | 0.017 | 0.017 |
| C(33) | 0.024 | 0.024 | 0.024 |
| N(34) | 0.007 | 0.007 | 0.007 |
| C(35) | 0.023 | 0.023 | 0.023 |
| N(36) | 0.006 | 0.006 | 0.006 |
| C(37) | 0.028 | 0.028 | 0.028 |
| C(38) | 0.019 | 0.019 | 0.019 |
| C(39) | 0.015 | 0.015 | 0.015 |
| C(40) | 0.023 | 0.023 | 0.023 |
| C(41) | 0.015 | 0.015 | 0.015 |
| C(42) | 0.02 | 0.02 | 0.02 |
| C(43) | 0.016 | 0.016 | 0.016 |
| C(44) | 0.02 | 0.02 | 0.02 |
| C(45) | 0.027 | 0.027 | 0.027 |
| C(46) | 0.015 | 0.016 | 0.015 |
| C(47) | 0.029 | 0.029 | 0.029 |
| C(48) | 0.017 | 0.017 | 0.017 |
| Au(49) | 0.068 | 0.067 | 0.067 |

**Table S4.** Adsorption energy of fentanyl on Au@S-rGO surface

| E(Au@S-rGO-fentanyl) | E(Au@S-rGO) | E(fentanyl) | ∆E (Ha) | ∆E (Kcal/mol) |
| --- | --- | --- | --- | --- |
| -4345.795732 | -3305.903889 | -1039.843344 | -0.048499 | -30.43 |

**References**

[1] R. K. Mishra, K. Y. Goud, Z. Li, C. Moonla, M. A. Mohamed, F. Tehrani, H. Teymourian, J. Wang, *J. Am. Chem. Soc.* **2020**, 142 (13), 5991.

[2] S. A. Goodchild, L. J. Hubble, R. K. Mishra, Z. Li, K. Y. Goud, A. Barfidokht, R. Shah, K. S. Bagot, A. J. S. McIntosh, J. Wang, *Anal. Chem.* 2019, 91 (5), 3747.

[3] E. Naghian, E. Marzi Khosrowshahi, E. Sohouli, F. Ahmadi, M. Rahimi-Nasrabadi, V. Safarifard, *New. J. Chem.* **2020**, 44 (22), 9271.

[4] E. Sohouli, A. H. Keihan, F. Shahdost-fard, E. Naghian, M. E. Plonska-Brzezinska, M. Rahimi-Nasrabadi, F. Ahmadi, *Mater. Sci. Eng. C. Mater. Biol. Appl.* **2020**, 110, 110684.

[5] A. Barfidokht, R. K. Mishra, R. Seenivasan, S. Liu, L. J. Hubble, J. Wang, D. A. Hall, *Sens. Actuators. B. Chem.* **2019**, 296, 126422.

[6] N. Wester, E. Mynttinen, J. Etula, T. Lilius, E. Kalso, B. F. Mikladal, Q. Zhang, H. Jiang, S. Sainio, D. Nordlund, E. I. Kauppinen, T. Laurila, J. Koskinen, *ACS Appl. Nano. Mater.* **2020**, 3 (2), 1203.

[7] Z. Zhao, Y. He, X. Qi, N. Li, Z. He, B. Liu, H. Lai, Y. Chen, T. Jin, *Electrochim. Acta.* **2023**, 462, 142747.

[8] M. Li, H. Chen, A. Xu, S. Duan, Q. Liu, R. Zhang, S. Wang, H. Bai, *Anal. Chim. Acta.* **2024**, 1312, 342686.

[9] M. Li, S. Duan, H. Chen, F. Zou, G. Zhang, Q. Liu, R. Zhang, X. Zeng, H. Bai, *Mikrochim. Acta.* **2023**, 190 (10), 414.
